# Susceptibility of Primary HTLV-1 Isolates from Patients with HTLV-1-Associated Myelopathy to Reverse Transcriptase Inhibitors

**DOI:** 10.3390/v3050469

**Published:** 2011-05-05

**Authors:** Beatrice Macchi, Emanuela Balestrieri, Arianna Ascolani, Silva Hilburn, Fabiola Martin, Antonio Mastino, Graham P Taylor

**Affiliations:** 1 Department of Neuroscience, University of Rome “Tor Vergata”, Via Montpellier 1, 00133, Rome, Italy; E-Mails: macchi@med.uniroma2.it (B.M.);balestrieri@med.uniroma.it (E.B.);ascolaria@hotmail.it (A.A.); 2 Department of Communicable Diseases, Faculty of Medicine, Imperial College London, Norfolk Place, London W2 1PG, UK; E-Mail: s.hilburn@imperial.ac.uk (S.H.); 3 National Centre for Human Retrovirology, St. Mary’s Hospital, Imperial College Healthcare NHS Trust, Praed St, London W2 1NY, UK; E-Mail: fabiola.martin@hyms.ac.uk (F.M.); 4 Department of Life Sciences, Section of Microbiological, Genetic and Molecular Sciences, University of Messina, Via F. Stagno d’Alcontres 31, 98166, Messina, Italy; E-Mail: antonio.mastino@unime.it (A.M.); 5 IRCCS, Santa Lucia, Via Ardeatina 306, 00179, Rome, Italy; 6 IRCCS Centro Neurolesi “Bonino- Pulejo”, Via Provinciale Palermo, 98124 Messina, Italy

**Keywords:** Human T-cell Lymphotropic Virus type 1, primary isolates, reverse transcriptase, drug sensitivity, azidothymidine, lamivudine, tenofovir, phosphonated carbocyclic 2′-oxa-3′aza nucleosides

## Abstract

Since human T-lymphotropic virus type 1 (HTLV-1)-associated diseases are associated with a high HTLV-1 load, reducing this load may treat or prevent disease. However, despite *in vitro* evidence that certain nucleoside/nucleotide analogue reverse transcriptase inhibitors (NRTIs) are active against HTLV-1, *in vivo* results have been disappointing. We therefore assayed the sensitivity of HTLV-1 primary isolates to a panel of RT inhibitors. HTLV-1 primary isolates were obtained, pre- and post- NRTI treatment, from patients with HTLV-1-associated myelopathy. Sensitivity to azidothymidine (AZT), lamivudine (3TC), tenofovir (TDF) and three phosphonated carbocyclic 2’-oxa-3’aza nucleosides (PCOANs) was assessed in a RT inhibitor assay. With the exception of 3TC, HTLV RT from primary isolates was less sensitive to all tested inhibitors than HTLV-1 RT from MT-2 cells. HTLV-1 RT from primary isolates and from chronically infected, transformed MT-2 cells was insensitive to 3TC. Sensitivity of primary isolates to RT inhibitors was not reduced following up to 12 months of patient treatment with AZT plus 3TC. The sensitivity of HTLV-1 primary isolates to NRTIs differs from that of cell lines and may vary among patients. Failure of NRTIs to reduce HTLV-1 viral load *in vivo* was not due to the development of phenotypic NRTI resistance. AZT and the three PCOANs assayed all consistently inhibited primary isolate HTLV-1 RT.

## 1. Introduction

Human T-lymphotropic virus type 1 (HTLV-1) is the causative agent of HTLV-1-associated myelopathy (HAM) [1]. This chronic debilitating disease is characterized by progressive spastic paraparesis, urinary, bowel and sexual dysfunction, and pain. HAM is reported to occur in 0.25% of HTLV-1 carriers in Japan [2] and is 10-fold higher among HTLV-1 carriers elsewhere [3]. Compared to HTLV-1 infected asymptomatic carriers, patients with HAM have ten-fold higher HTLV-1 proviral load [4] and this has been reported to predate clinical disease [5].

### 1.1. In vitro RT Inhibition

Like the human immunodeficiency viruses (HIV-1 and HIV-2), HTLV-1 encodes reverse transcriptase (RT), integrase and protease enzymes that are potential therapeutic targets. *In vitro* studies have shown that the thymidine analogue RT inhibitor azidothymidine (AZT) prevented HTLV-1 infection [6]. In contrast, the inability of nucleoside RT inhibitor lamivudine (3TC) to inhibit HTLV-1 cell-to-cell transmission [7] has been ascribed to a “natural resistance” of the HTLV-1 RT to 3TC [8,9]. Mutations at codons 151 and 184, which have been well characterized in HIV-1 drug resistance, have not been implicated in HTLV resistance [8]. Whilst a V-I polymorphism at codon 118 has been found [9] it is noted that other retroviruses also exhibit natural resistance to 3TC. In addition, pre-activated triphosphorylated AZT inhibited HTLV-1 RT *in vitro* [8,10] at much lower concentrations than pre-activated 3TC using various cell-free RT assays [8,11]. The adenine nucleotide analogue, 9-(R)-[2-(phosphonomethoxy)propyl] adenine (tenofovir disoproxil fumarate, TDF) prevents HTLV-1 transmission *in vitro* and inhibits the enzymatic activity of HTLV-1 RT extracted from the chronically HTLV-1 infected MT-2 cell line at lower concentrations than AZT [12]. A new class of phosphonated nucleotide analogues, the carbocyclic 2’-oxa-3’-aza-nucleosides (PCOANs) are endowed with a phosphate group heavily attached to the acyclic nucleoside moiety, and have recently been shown to inhibit HTLV-1 RT activity and to prevent HTLV-1 transmission *in vitro* [10,13].

### 1.2. In vivo RT Inhibition

AZT has been shown to protect rabbits from challenge with HTLV-1 infection [14], while the cytosine analogue 3TC was reported to reduce HTLV-1 viral load in a small open study of patients with HAM [15]. Between 1999 and 2002, sixteen patients with HAM participated in a randomized, double blind, placebo controlled study of AZT plus 3TC, the Bridge Study. After six months, as per study design, all patients continued with open-label AZT and 3TC. Thus, eight patients took placebo for six months followed by the active compounds for six months, and eight patients took AZT and 3TC for twelve months. Peripheral blood mononuclear cells (PBMCs) were viably preserved at four to eight week intervals. No significant change in HTLV-1 viral load was found up to 48 weeks of therapy with this combination, nor was there any improvement in immunological or clinical parameters [16].

*In vitro* data had indicated that tenofovir, which has an established safety profile, inhibits HTLV-1 RT better than other licensed RT inhibitors [17]. Thus, six patients attending the HTLV clinic were treated, after informed consent, off-license with 245 mg tenofovir daily for a mean of 8.7 (± 2.3) months. As presented below, no effect on HTLV-1 viral load was seen during treatment with tenofovir. Therefore, in addition to testing for phenotypic resistance to AZT and 3TC, the primary isolates from patients in the Bridge Study were also examined for phenotypic evidence of susceptibility to tenofovir and the three novel RT inhibitors.

Here we present data on the *ex vivo* inhibition of HTLV-1 RT isolates from patients who participated in the Bridge study pre and post therapy. We demonstrate that the NRTI sensitivity of HTLV-1 RT derived from cell lines differs from that of primary isolates, and show differences in drug susceptibility between primary isolates.

## 2. Results and Discussion

### 2.1. RT Activity in Isolates from both HAM Patient PBMC Cultures and from the HTLV-1 Chronically Infected MT-2 Cell Line

**Figure 1:**
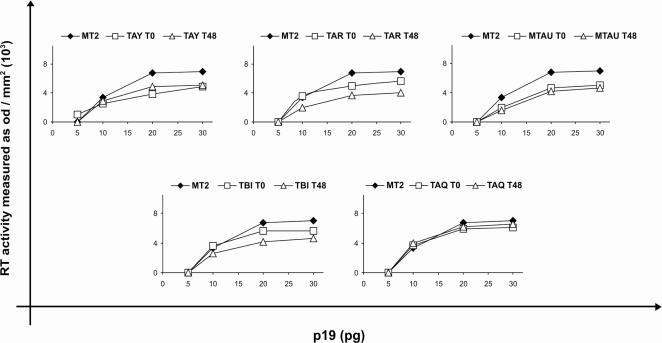
RT activity in human T-lymphotropic virus type 1 (HTLV-1) isolates from peripheral blood mononuclear cell (PBMC) cultures of HTLV-1-associated myelopathy (HAM) patients. Centrifuge-enriched isolates from PBMC cultures of 5 patients with HAM (TAY, TAR, TAU, TBI, TAQ), before (T0) and after (T48) therapy with 3TC plus AZT, or from MT-2 cells, were assayed for p19. Serial dilutions of p19-equivalent isolates were added to the RT assay and amplified products of the template were visualized on a 2% agarose gel and quantitated by densitometry, as a measure of RT activity.

To test the *in vitro* inhibitory activity of different compounds on HTLV-1 RT from patient isolates, the optimal amount of RT needed to generate cDNA from a known RNA template was first assessed in centrifugation-enriched isolates from patient PBMC culture and, for comparison, from MT-2 cell cultures. Isolates were prepared from PBMC cultures of all of the patients under study collected before and after 48 weeks of therapy with AZT plus 3TC except for patient TBJ, due to insufficient material. The amount of HTLV-1 p19 in the enriched isolates was quantified as a surrogate measure of the amount of RT. Serial dilutions of resuspended isolates, corresponding to 5 to 30 pg of p19, were added to the assay mixture in order to determine the amount of p19-equivalent HTLV-1 RT to be used in the assay. The amount of amplified product plateaued with the addition of isolate preparations corresponding to 20–30 pg of p19 (Figure 1). These results indicated that the utilized assay was sensitive enough to measure RT activity in PBMC cultures from HTLV-1 infected patients and that isolates from HAM patient PBMC cultures, before and after therapy, and from MT-2 cells plateaued for RT activity at similar p19-equivalent concentrations. Based on these results, supernatants containing the equivalent of 24 pg of p19 were used in all the subsequent inhibitory assays.

### 2.2. Sensitivity of Patient HTLV-1 Isolates to RT Inhibitors

The concentration of AZT, 3TC, TDF and of PCOAN1, PCOAN2, PCOAN3 required to inhibit RT activity in primary isolates prior to and after any antiretroviral therapy was determined. The concentrations used are shown in Table 1. HTLV-1 RT from these PBMC culture derived primary isolates was inhibited by AZT (4–8 nM), which was ten-times more than the concentration required to inhibit RT from MT-2 cells (Table 2). In samples from five out of six patients and from MT-2 cells the inhibitory concentration of 3TC was >10^3^ nM but in one patient (TBJ) the inhibitory concentration of 3TC was 400 nM. HTLV-1 RT from MT-2 cells was susceptible to TDF at 0.4 nM but 10–1000 nM of this compound were required to inhibit RT from the primary isolates. The three unlicensed compounds all inhibited MT-2 HTLV-1 RT at 0.8 nM and also inhibited HTLV-1 RT from the primary isolates at 2–10 nM, with PCOAN1 being the most potent. No change in the inhibitory concentration of any RT inhibitor was seen in the primary isolates obtained from patients after 6 to 12 months therapy with AZT plus 3TC.

### 2.3. Virus Release in Cultures of PBMCs from Patients with HAM

**Figure 2:**
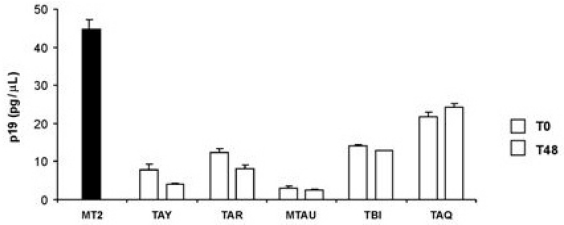
p19 release in culture supernatants from PBMCs of HAM patients before and after therapy. The p19 concentration in supernatants from PBMC cultures of 5 patients with HAM (TAY, TAR, TAU, TBI, TAQ), before (T0) and after (T48) therapy with 3TC plus AZT, was evaluated by ELISA. Values are expressed as the mean of quadruplicate samples ± standard deviation. Production of p19 (black column) in the supernatant of chronically infected cells MT-2 was used as a positive control.

The supernatants utilized for obtaining centrifugation-enriched isolates to assay, were also evaluated for viral particle production by measuring released p19. Figure 2 shows that MT-2 cells produced more p19 (40 pg/μL) than did cultures of PBMCs from HAM patients (5–20 pg/μL). However the amount of p19 in supernatants from PHA stimulated PBMCs from patients with HAM was similar before and after 48 weeks of antiretroviral treatment.

### 2.4. Proviral Load and pol Sequence

*In vivo* HTLV-1 DNA loads and the duration of exposure to nucleoside analogues are presented in Table 2. As previously reported, no significant change in HTLV-1 proviral load was seen *in vivo* after 24–48 weeks of therapy with AZT plus 3TC [16]. In our samples the median viral load was 3.0 copies per 100 PBMCs before therapy, and 3.05 copies per 100 PBMCs after therapy. To determine whether the lack of reduction in proviral load was due to mutations in the HTLV-1 reverse transcriptase, proviral *pol* was sequenced at baseline and at the conclusion of therapy. In three patients the consensus sequence was wild type at both time points. Conversely, duplications, insertions or deletions were observed in the other three patients. However, no relationship was observed between individual proviral load variations and detection of mutations in patients. Thus, apparently the lack of reduction in proviral load was not due to the outgrowth of mutated viruses.

### 2.5. In vivo Exposure to Tenofovir

**Figure 3:**
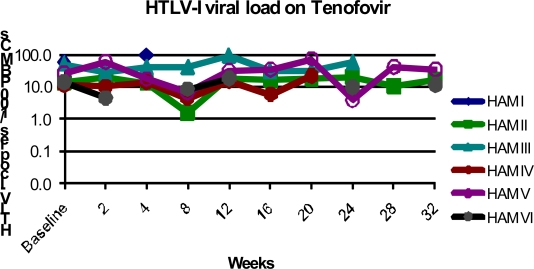
*In vivo* HTLV-1 viral load (HTLV DNA copies /100 PBMCs) during treatment with tenofovir.

TDF was well tolerated with no reported side effects. Whilst some patients exhibited a deterioration during therapy those patients who received therapy for longest had measurable improvement in pain and/or gait (Tables 3 and 4). Total lymphocyte count increased during treatment with TDF due to increases in both CD4+ and CD8+ lymphocytes. However, no significant change in HTLV-1 viral load was seen during up to 16 months therapy with TDF (Figure 3). The median baseline HTLV viral load was 15.2 HTLV-1 DNA copies /100 PBMCs compared to 19.6 HTLV-1 DNA copies /100 PBMCs after therapy with TDF.

## 3. Experimental Section

### 3.1. Materials and Patients

In addition to the published ‘Bridge Study’, blood samples were collect during a study of the pathogenesis and treatment of HTLV-1 between April 2004 and May 2005, from five patients with HAM and one diagnosed with HTLV-1-associated encephalitis. The study was approved by the local research ethics committee. These patients had been, after informed consent and on a case-by-case basis, treated with TDF based on *in vitro* HTLV-RT data [12,17]. At the initiation of TDF treatment their mean age was 44.8 (± 15) and the mean duration of HAM was 8.3 (± 6.8) years. One patient was co-infected with HBV (HBeAg+). Four patients were female and three West African. Heterosexual intercourse was the most common route of HTLV-1 acquisition. Routine clinical and laboratory data were collected and pre and post-treatment values analyzed by T-test.

### 3.2. Cell Culture and Isolate Preparation

PBMCs from heparinized blood of patients with HAM were separated by Ficoll/Hypaque density gradient (Pharmacia Uppsala, Sweden) and then washed three times in phosphate buffered saline (PBS). To use virus isolates from patients as a source of RT for the subsequent assay, paired samples of PBMC at week 0 and week 48 from six patients were cultured at 0.8 × 10^6^ cells/mL in RPMI plus 15% FBS in the presence of 20 U/mL of IL-2, following a two-day stimulation with PHA. PBMCs from patients were maintained in culture for 3–4 weeks. PBMCs were pelleted weekly, supernatants harvested and fresh medium completely replenished. Aliquots of the harvested supernatants were utilized to detect viral particle production by p19 release, while the rest were cleared at 1,000 g and then ultracentrifuged at 30,000 g for 4 h at 4 °C. HTLV-1 p19 viral protein in centrifugation-enriched viral lysates was detected using a commercially available ELISA (see Section 3.6).

### 3.3. HTLV-1 Proviral Load

HTLV-1 proviral load in the patients treated with TDF was measured by quantitative real-time PCR as previously reported [18]. Samples were available at baseline, after 2 weeks and at 4 weekly intervals out to a maximum of 32 weeks.

### 3.4. In vitro Assayed Compounds

Three licensed reverse transcriptase inhibitors (AZT, 3TC and TDF) and three experimental compounds prototype PCOANs referred to as, PCOAN1, PCOAN2 and PCOAN3 were used. The acyclic phosphonate TDF was kindly provided by Dr. Jan Balzarini (Rega Institute for Medical Research, Leuven, Belgium) while AZT and 3TC were provided by Wellcome Research Laboratories (Beckenham, England, UK). The PCOANs are a family of newly synthesized compounds which belong to a group of cyclic nucleoside phosphonates, with the furanose ring replaced by an N,O-heterocyclic ring [19,20]. PCOANs were dissolved in DMSO and maintained at stock concentrations of 200 mM at −20 °C. TDF, AZT and 3TC were dissolved in culture medium without serum and maintained at stock concentrations of 100 mM at −20 °C.

### 3.5. HTLV-1-RT Activity Assay

To test the sensitivity of HTLV-1 isolates from patients to RT inhibitors we set up a novel *ex vivo*, cell-free HTLV-1-RT activity assay. This assay is a modified version of a method we have described and used to screen the HTLV-1-RT-inhibitory activity of antiretroviral compounds *in vitro* [10,12]. The modification essentially consists of using patient isolates, prepared as described in Section 3.2, as source of HTLV-1 RT in place of viral lysates from HTLV-1 chronically infected cell lines. RNA template to be subjected to reverse transcription into cDNA, was obtained from stably transfected cells ectopically expressing the herpes simplex virus type 1 glycoprotein D (gD) gene [21] and treated with RNase free DNase.

Compounds to which sensitivity was assayed were activated by pre-incubating with a crude extract, prepared from PHA plus IL-2 stimulated PBMCs from healthy, HIV/HBV/HCV negative donors. To prepare the crude extract, 1 × 10^6^ PBMCs, previously stimulated with PHA (2 μg/mL) and IL-2 (20 U/mL), for 72 h in RPMI plus 20% FCS, were lysed on ice in buffer (50 mM Tris-HCl pH 7.4, 1 mM EDTA, 1 mM EGTA pH 7.4, 0.05% Triton-X, 150 mM NaCl, 0.25% sodium deoxycholate, 0.1% NP-40 and, freshly added, 1 mM PMSF, 15 μM DTT, 5 μg/mL leupeptin, 5 μg/mL pepstatin, 5 μg/mL aprotinin, 1 mM Na_3_VO_4_, 20 mM Na_3_F, all from Sigma), and centrifuged at 10000 g. Lysed extracts were incubated with different compound concentrations for 15 minutes on ice followed by 45 minutes at 30 °C and then a 5 minute incubation at 95 °C to inactive the mixture. As an *ex vivo* source of HTLV-1 RT, viral lysates from different HAM patient sample isolates (24 pg of p19-equivalent material) were used for each RT-PCR reaction. Total DNase-treated RNA (1 μg) was specifically reverse transcribed using 0.5 μM reverse primer specific for the gD gene U_S_6 (5’ TGTCGTCATAGTGGGCCTCCAT 3’) in a reaction mix containing 1 × RT buffer, 100 U RNase inhibitor, 1 mM dNTP, 10 mM DTT, (all from Promega, Madison, WI, USA) plus HTLV-1-RT preparations from patients in a volume of 10 μL. The reactions were performed in the presence or absence of activated substances, for 1 h at 37 °C. After incubation at 95 °C for 5 minutes, DNA PCR (20 cycles) was performed using 5 μL of RT reaction in a mix containing 1X Taq Gold buffer (Promega), 0.5 μM primer pair (U_S_6 reverse, see above, and U_S_6 forward, 5′ AGACTTGTTGTAGGAGCATTCG 3′), 0.2 mM dNTP, 5 mM MgCl_2_, 1.25 U Taq Gold Polymerase (Promega). To investigate the dose–response, the assayed compounds, AZT, 3TC, TDF or PCOAN1, PCOAN2, PCOAN3 were added at the concentrations shown in Table 1. Amplified DNA (350 bp) was visualized on a 2% agarose gel containing ethidium bromide. HTLV-1 RT preparations from cultured MT-2 cells were used as a control.

### 3.6. Detection of p19 through Antigen Capture Assay

p19 in supernatants or enriched isolates was assayed using an HTLV-1 p19 Gag antigen capture enzyme-linked immunosorbent assay (ELISA) (ZeptoMetrix, Buffalo, NY, USA). Assays were performed according to the manufacturer’s protocols.

### 3.7. Genotypic Analysis of HTLV-1 pol Gene

Genotypic analysis of the entire *pol* coding region corresponding to nucleotides 2100–5076 of HTLV-1 reference sequence (NC_001436.1) was performed by sequencing PCR products from DNA of MT-2 cells and of cultured cells from patients enrolled in the study at baseline and at week 48 (post AZT and 3TC therapy). Primer pairs were designed to amplify overlapping products of about 300 nucleotides in length.

## 4. Conclusions

The sensitivity of HTLV-1 primary isolates to AZT, 3TC, TDF and three novel PCOANs has been determined using a novel RT inhibition assay. Using PBMCs from patients with HAM who had been treated for up to 48 weeks with AZT and 3TC, we have demonstrated for the first time, both the sensitivity of HTV-1 RT to AZT and its primary resistance to 3TC in HTLV-1 clinical isolates. Furthermore we have shown that prolonged *in vivo* treatment with AZT and 3TC was not associated with the development of reduced sensitivity of HTLV-1 RT to these nucleoside analogues or with consistent changes in the sequence of RT. Therefore, we conclude that the lack of viral, and consequent lack of immunological and clinical response seen in this study [16] was not due to the development of genotypic or phenotypic resistance. Since the proviral *pol* consensus sequence was identical to the reference sequence in 3/6 patients these results will likely be applicable to the majority of patients infected with HTLV-1.

Unexpectedly, the HTLV-1 RT from patients’ PBMCs displayed considerable variability in TDF sensitivity and consistent sensitivity to PCOANs. In the present study, the phenotypic RT sensitivity of whole virus primary isolates from patients with HAM was quantitatively similar in samples from untreated *versus* RT inhibitor treated patients, and at least one order of magnitude, and in some cases a thousand times, less sensitive to AZT and to TDF than RT from MT-2 cells. This lack of sensitivity to TDF was seen prior to, and not altered by, *in vivo* NRTI exposure. We have not, at the moment, a clear explanation for the different sensitivity of isolates from patients and MT-2 associated virus to RT-inhibitors. However, we have previously demonstrated variable efficacy of RT inhibitors against HTLV-1 RT from different transformed cell lines. For example, the concentration of PCOAN2 needed to inhibit RT activity from C5/MJ and MT-2 cell supernatants was 10–100 times less than that required to inhibit RT from C91/PL cells. Similarly, the concentration of TDF needed to inhibit RT from MT-2 cell supernatants was 10–100 times less than that required to inhibit RT from C91/PL and C5/MJ cells. Conversely, RT from HUT 102 supernatants was resistant to most of the RT inhibitors used [13]. Whereas, a study in which RT produced only from an infectious HTLV-1 molecular clone derived from CS-1 cells (HTLV-1 transformed B cell line) was utilized, concluded that HTLV-1 was highly sensitive to TDF [17]. Thus, phenotypic sensitivity of HTLV-1 RT to inhibitors seems a phenomenon much more variable than expected by genotypic relatively low variability of the virus. Further study is needed on this aspect.

While the data from this study do not suggest that a combination of AZT with TDF might inhibit HTLV-1 RT in some patients, this possibility remains. We therefore promote pre-treatment phenotypic sensitivity testing to avoid unnecessary exposure to these therapies. Conversely, each of the PCOANs assayed had an RT inhibitory concentration similar to that of AZT suggesting that these compounds should be considered in future studies. We have recently demonstrated that these new RT inhibitors, PCOANs, are able to inhibit HTLV-1 cell-to cell transmission *in vitro* using MT-2 and HTLV-1 donor cells as a source of HTLV-1 as effectively as TDF and AZT [13].

The presence of defective HTLV-1 proviruses in clinical samples is well known [22] and our findings here, together with recent data on the number and size of clonally expanded HTLV-1 infected cells *in vivo* [23], suggest that the consensus sequencing has, in some patients, amplified DNA from dominant clones that may not be replication competent. However, the persistent phenotypic sensitivity of RT from HTLV-1 isolates to AZT, including those cases in which mutated/deleted forms of proviral *pol* were detected, suggests that the lack of virological effect in the dual therapy clinical trial was not due to the emergence of mutations in RT during therapy. This finding supports the hypothesis that RT only partially explains HTLV-1 viral load maintenance and spread within patients with HAM while mitosis plays a major role in maintaining HTLV-1 viral burden. It is less likely that viral load was maintained by HTLV-1 infection in drug sanctuary sites with migration of infected lymphocytes into the peripheral blood.

Overall, patients treated with TDF showed no clinical improvement. The apparent association between longer duration of TDF therapy and reduced pain and 10m timed walk most likely reflects a bias to continue therapy due to perceived benefit. The CD4+ and CD8+ T lymphocyte count rose significantly from baseline. In HIV infection a rise in CD+ T lymphocyte count is associated with a decreased HIV RNA viral load in patients treated with TDF and other antiretroviral therapies. We could not find previous reports of a change in lymphocyte counts in HIV or HTLV-1 non-infected patients treated with antiretrovirals. In HTLV-1 infected patients AZT did not cause a significant rise of lymphocyte counts. In our earlier study of patients with HAM receiving dual-therapy with AZT and 3TC, the CD4+ T lymphocyte counts rose, but the total lymphocyte count and the CD8+ T lymphocyte count fell. In the same study total lymphocyte and subset counts increased in the placebo group at week 24 but by much less than seen in patients treated with TDF. We hypothesize that, in addition to the antiretroviral effect, TDF therapy is associated with a virus independent increase in lymphocyte and T cell subset counts.

Is there any role for HTLV-1 RT inhibitors in clinical practice? Treatment of adult T-cell leukaemia/lymphoma with AZT together with interferon-α currently induces remission in about 70% of patients. However, there is no evidence that the available RT inhibitors, given as monotherapy or in combination with other RT inhibitors, reduce HTLV viral load. Recently, AZT in combination with a histone deacetylase inhibitor significantly and persistently reduced STLV-1 viral load in naturally infected baboons over three months of treatment [24] and reproduction of this effect in HTLV-1 infection *in vivo* is urgently required.

If sustained reduction in HTLV-1 viral load in patients with, or at risk of, HAM requires long term combination therapy that includes an RT inhibitor, less toxic alternatives to AZT will be desirable. TDF, which is better tolerated than AZT, would be a suitable candidate based on data using MT-2 cells as donor cells or as a source of RT. However, our data suggest that TDF might not be as effective as AZT *in vivo*, due to its reduced sensitivity in treating naïve HTLV-1 primary isolates. Thus, alternative HTLV-1 inhibitors should be tested.

Since HTLV-1 is a highly cell-associated virus, RT inhibitors might be useful in clinical trials when used in combination with compounds such as cell signaling modulators [25]. Therefore, predicting the sensitivity of HTLV-1 isolates from patients to different RT inhibitors using our *ex vivo* cell-free assay may be particularly useful in preclinical studies for future clinical trials.

While HTLV-1 RT inhibitors are not routinely used clinically to reduce the risk of transmission following occupational exposure or following delivery by an HTLV-1 infected mother, they remain the only potential therapies for pre- or post- exposure prophylaxis. The data presented here suggest that AZT rather than TDF (unless sensitivity is known) would be the preferred therapeutic agent following high-risk exposure. The lack of efficacy in the six patients studied, in conjunction with the *ex vivo* data from an additional six patients who were not exposed to TDF, suggests that reduced sensitivity may be common but this needs to be confirmed with more HTLV-1 primary isolates. These data suggest a general lack of RT efficacy when used alone as an anti-HTLV therapy in patients with HAM, justify further studies of novel drug combinations and/or PCOANs to treat and prevent HTLV-1 infection, and provide a tool to test HTLV-1 RT susceptibility to trial drugs *ex vivo*.

## Figures and Tables

**Table 1:** Compounds and concentrations.

**Compound**	**Concentrations in nM**					
Azidothymidine	10	8	6	4	2	1
Lamivudine	1000	800	600	400	200	100
Tenofovir df	1000	100	10	1	0.1	
PCOAN1	10	8	6	4	2	1
PCOAN2	10	8	6	4	2	1
PCOAN3	10	8	6	4	2	1

**Table 2:** *In vitro* human T-lymphotropic virus type 1 (HTLV-1) RT inhibitory concentrations (nM).

**Patient Time Point**	**Duration of AZT/3TC**	**HTLV-1 DNA Copies/ 100 PBMCs**	**AZT**	**3TC**	**TDF**	**PCOANs**			**Mutations Detected by HTLV-1 Proviral-DNA *pol* Gene Sequence Analysis Compared with Reference Sequence—Accession Number NC_001436.1**
						1	2	3	
TAY									
Week 0		1.0	6	>10^3^	10	2	6	4	None detected
Week 48	24 weeks	4.8	6	>10^3^	10	2	6	4	None detected
TAR									
Week 0		4.3	6	>10^3^	10^2^	4	10	6	None detected
Week 48	48 weeks	0.6	6	>10^3^	10^2^	4	10	6	12 nt duplication at position 3551
TAU									
Week 0		2.0	6	>10^3^	10	4	10	8	None detected
Week 48	24 weeks	1.0	6	>10^3^	10	4	8	8	None detected
TBI									
Week 0		3.4	6	>10^3^	10	6	10	6	None detected
Week 48	48 weeks	17.7	6	>10^3^	10	8	10	6	None detected
TBJ									
Week 0		6.3	4	4^*^10^2^	10^2^	8	10	10	2 nt insertion at position 2935
Week 48	48 weeks	8.1	Insufficient material						
TAQ									
Week 0		2.6	8	>10^3^	10^3^	8	10	8	102 nt deletion at position 4031
Week 48	24 weeks	1.3	8	>10^3^	10^3^	8	10	8	75 nt deletion at position 4102
MT-2			0.4	>10^3^	0.4	0.8	0.8	0.8	None detected

**Table 3:** Clinical, virological and immunological outcomes of patients with HAM receiving tenofovir (TDF) at baseline and at end of treatment.

		**TDF Started (%, ±SD)**	**TDF Stopped (%, ±SD)**	**Mean Difference (SD)**	***P***
Timed walk	Mean	14.2 (±3.5)	16.1 (±2.6)	+1.9 (±2.7)	0.4
	Median	16.7	15.8		
Visual analogue pain score	Mean	4.1 (±1.7)	4.7 (±2)	+0.6 (±1.3)	0.3
	Median	7	7.5		
HTLV-I proviral load (copies/100 PBMCs)	Mean	39.8 (±14)	38.9 (±13)	−0.9 (±12.7)	0.9
	Median	15.2	19.6		
Lymphocyte count (10^9^/L)	Mean	2320 (±1070)	2882 (±1395)	+562 (±575)	0.07
	Median	1800	2710		
CD4+ T lymphocyte (10^9^/L)	Mean	962 (±567)	1218 (±640)	+256 (±582)	0.35
	Median	840	1390		
CD8+ T lymphocyte (10^9^/L)	Mean	620 (±399)	774 (±529)	+154 (±546)	0.9
	Median	530	560		

**Table 4:** Percent change in clinical and laboratory parameters when tenofovir treatment was stopped compared to baseline values.

**Patient**	**Duration of Treatment (Months)**	**TW (%)**	**VAS (%)**	**VL (%)**	**VL Log Change**	**Total Lymphocyte Count (%)**	**CD4+ (%)**	**CD8+ (%)**
**I**	2.08	wc	Nd	−1.5	−0.007	15	nd	nd
**II**	2.55	−4.7	0	312.3	−0.23	−16.7	−60.7	−52.8
**III**	8.33	2.6	36.4	−16.1	0.12	33.3	36.4	15.2
**IV**	9.49	11.1	25	11.9	0.62	8.1	18.6	−12.5
**V**	12.26	−23.1	0	−40.6	−0.08	0	414.8	325.8
**VI**	16.56	−13.3	−14.3	28.4	0.05	43.8	8.3	5.4

TW = timed walk, VAS = visual analogue pain score, VL = HTLV-I viral load, wc = wheelchair, nd = not done.
